# Impacto dos Atrasos de Reperfusão e das Desigualdades Interseccionais no Cuidado ao IAMCSST no Rio de Janeiro

**DOI:** 10.36660/abc.20250861

**Published:** 2026-03-09

**Authors:** Henrique Tria Bianco

**Affiliations:** 1 Universidade Federal de São Paulo Escola Paulista de Medicina São Paulo SP Brasil Escola Paulista de Medicina da Universidade Federal de São Paulo (EPM/UNIFESP), São Paulo, SP – Brasil

**Keywords:** Infarto do Miocárdio com Supradesnível do Segmento ST, Equidade em Saúde, Reperfusão

## Introdução

Encontra-se bem estabelecida na literatura a importância da reperfusão precoce nos quadros de infarto agudo do miocárdio com supra ST (IAMCSST).^1,2^ Apesar das melhorias no cuidado, até um terço dos pacientes que apresentam esse evento dentro de 12 horas após o início dos sintomas ainda não recebem terapia de reperfusão aguda. A intervenção coronária percutânea primária (ICPp), quando realizada com celeridade em centros de alto volume e qualificados, tende a superar a fibrinólise. Contudo, a fibrinólise pode ser a estratégia inicial mais adequada para muitos pacientes. Como até 70% dos casos de IAMCSST chegam a hospitais sem ICPp disponível e os tempos porta-balão frequentemente se prolongam devido a atrasos de transporte, o benefício da ICP pode ser atenuado, o que ressalta o papel, por vezes subestimado, da fibrinólise precoce.^3,4^ Ao selecionar a estratégia de reperfusão, é imprescindível considerar as limitações logísticas (triagem e transporte), lembrando que a fibrinólise rápida pode ser superior quando o atraso adicional até a ICP excede cerca de 1 hora. A atenção rigorosa a algumas variáveis é crucial para garantir reperfusão segura e rápida, especialmente no ambiente pré-hospitalar.^3,5^ Entretanto, diversidades clínicas e epidemiológicas como as observadas no Brasil, podem impactar nos desfechos relevantes.^6–8^

Diante desse cenário paradoxal — no qual a rapidez da reperfusão define o prognóstico, mas persistem atrasos e desigualdades no acesso — o estudo EQUITY-MI (*EQuity, Universality and Intersectionality: the challenge of ThrombolYsis in patients with Myocardial Infarction*) foi desenvolvido para avaliar a equidade no atendimento ao IAMCSST no Rio de Janeiro. Ao oferecer um retrato da aplicação das políticas vigentes e de seu impacto real no sistema de saúde, propôs identificar gargalos processuais, diferenças por território, gênero e raça/cor, na expectativa de ampliar a reperfusão precoce e promover equidade de resultados.^9^

Este interessante e necessário estudo, analisou 457 pacientes com infarto agudo do miocárdio (IAM) atendidos na Região Metropolitana do Rio de Janeiro, com foco no tempo porta-agulha (TPA) da trombólise e em possíveis desigualdades regionais. A análise contemplou perfis sociodemográficos, comorbidades, estratégia de reperfusão e desfechos de função ventricular.

### Perfil sociodemográfico e clínico

A amostra apresentou idade média de 60,4 anos, com predominância do sexo masculino (72,7%). Observou-se marcante composição racial com maioria de pessoas negras, escolaridade concentrada no ensino médio (38,2%) e renda familiar entre um e dois salários mínimos (35,8%). Houve elevada carga de comorbidades: hipertensão arterial (64,7%), diabetes mellitus (32%) e tabagismo (34,4%). Esse perfil indica população com risco cardiovascular significativo e potencial vulnerabilidade socioeconômica, fatores que podem influenciar tanto o acesso quanto a qualidade do cuidado. Do ponto de vista territorial, as áreas de atendimento foram agrupadas em: Zona Norte/Ilha do Governador; Zona Sul/Grande Tijuca; Zona Oeste; Barra da Tijuca/Jacarepaguá; Centro; além das macrorregiões Baixada Fluminense e Leste Metropolitano.

### Estratégia de reperfusão e tempo porta-agulha

Quanto à reperfusão, 79% (n=363) receberam trombólise; 18,5% (n=85) não receberam; e 2,4% (n=11) não tinham registro. Entre os 363 trombolisados, 327 possuíam TPA registrado: a mediana foi de 77 minutos, e apenas 20,9% receberam trombólise em até 30 minutos. Este achado é clinicamente relevante: metas assistenciais usuais recomendam TPA inferior a 30 minutos, de modo que a mediana de 77 minutos sugere atrasos importantes e oportunidade de melhoria de processos, sobretudo nas etapas pré-hospitalares, triagem, diagnóstico eletrocardiográfico e logística de medicação.

### Resultados por região

No modelo linear generalizado (GLM), as médias ajustadas de TPA (min) foram: Baixada Fluminense 165,9; Barra/Jacarepaguá 124,2; Centro 67,7; Leste Metropolitano 123,5; Zona Norte 100,4; Zona Oeste 114,0; Zona Sul/Grande Tijuca 112,8. As estimativas de regressão quantílica reforçam gradientes:

- Q25 (atendimentos mais rápidos): ~32,0 min na Zona Sul/Grande Tijuca versus 52,0 min na Baixada Fluminense.
- Mediana (Q50): 74,9 min no Centro versus 122,9 min na Baixada Fluminense.
- Q75 (25% mais lentos): 100,3 min no Centro versus 243,1 min na Baixada Fluminense.

Tais diferenças, embora nem sempre estatisticamente significativas, são clinicamente expressivas e sugerem assimetrias operacionais e de acesso, sobretudo desfavoráveis à Baixada Fluminense. O desempenho do Centro, com tempos sistematicamente menores, pode refletir maior disponibilidade de recursos, protocolos mais ágeis ou melhor integração pré-hospitalar.

### Desfecho de função ventricular

Na alta hospitalar, 63,5% apresentaram algum grau de disfunção ventricular. Identificou-se tendência a maior proporção de disfunção grave na Barra/Jacarepaguá, sem atingir significância estatística. Ainda que exploratório, esse achado reforça a importância de processos de cuidado oportunos, dado o impacto do tempo de isquemia na função miocárdica.

### Comentários e implicações

- O baixo percentual de TPA ≤30 minutos (20,9%) e a mediana de 77 minutos apontam para a necessidade de reengenharia do fluxo: diagnóstico precoce (ECG rápido), protocolos de triagem com gatilhos de trombólise, preparo prévio de fármacos e treinamento de equipes.
- A idade associada a maiores TPA sugere que barreiras clínicas ou de avaliação de risco em pacientes mais velhos possam retardar decisões terapêuticas; intervenções específicas para esse grupo podem ser benéficas.
- As variações regionais, com pior desempenho na Baixada Fluminense, indicam desigualdades estruturais. Investimentos em rede de urgência, linhas de cuidado regionais, teleconsultoria para ECG e padronização de protocolos podem reduzir disparidades.
- A combinação de GLM e regressão quantílica é metodologicamente apropriada para dados assimétricos, permitindo captar diferenças tanto no centro quanto nas caudas da distribuição.

### Limitações (inferidas)

Registros incompletos (p.ex., TPA ausente em parte dos trombolisados e N reduzido no GLM) podem introduzir viés de seleção. A ausência de significância em algumas análises não exclui relevância clínica, especialmente ante tamanhos de efeito e gradientes observados.

## Conclusão

Observou-se uma alta carga de risco cardiovascular em uma população com vulnerabilidades socioeconômicas. Apesar da ampla utilização da trombólise, o TPA mostrou-se prolongado e heterogêneo entre regiões, com desempenho consistentemente inferior em áreas periféricas. A priorização de estratégias voltadas à equidade territorial é fundamental para melhorar desfechos, inclusive a função ventricular esquerda pós-evento agudo.
