# Supplementary material for: Impacto dos Atrasos de Reperfusão e das Desigualdades Interseccionais no Cuidado ao IAMCSST no Rio de Janeiro
Source: Arq Bras Cardiol. 2026 Mar 9;122(12):e20250861. [Article in Portuguese] doi: 10.36660/abc.20250861 (PMC13155785; doi:10.36660/abc.20250861)
Supplement: Translation

# Translation: Impact of Reperfusion Delays and Intersectional Inequalities in the Care of STEMI in Rio de Janeiro

**DOI:** 10.36660/abc.20250861i

**Authors:** Henrique Tria Bianco

**Affiliations:** 1 São Paulo SP Brazil Escola Paulista de Medicina da Universidade Federal de São Paulo (EPM/UNIFESP), São Paulo, SP – Brazil

**Keywords:** ST Elevation Myocardial Infarction, Health Equity, Reperfusion

## Introduction

The importance of early reperfusion in cases of ST-segment elevation myocardial infarction (STEMI) is well established in the literature.^1,2^ Despite improvements in care, up to one-third of patients who experience this event within 12 hours of symptom onset still do not receive acute reperfusion therapy. Primary percutaneous coronary intervention (PCI), when performed promptly in high-volume and qualified centers, tends to outperform fibrinolysis. However, fibrinolysis may be the most appropriate initial strategy for many patients. Since up to 70% of STEMI cases arrive at hospitals without available PCI, and door-to-balloon times are often prolonged due to transport delays, the benefit of PCI may be attenuated, highlighting the sometimes underestimated role of early fibrinolysis.^3,4^ When selecting a reperfusion strategy, it is essential to consider logistical limitations (triage and transport), remembering that rapid fibrinolysis may be superior when the additional delay to PCI exceeds approximately 1 hour. Rigorous attention to certain variables is crucial to ensure safe and rapid reperfusion, especially in the prehospital setting.^3,5^ However, clinical and epidemiological diversities, such as those observed in Brazil, can impact relevant outcomes.^6–8^

Given this paradoxical scenario—in which the speed of reperfusion defines the prognosis, but delays and inequalities in access persist—the EQUITY-MI study (Equity, Universality and Intersectionality: the challenge of ThrombolYsis in patients with Myocardial Infarction) was developed to evaluate equity in care for STEMI in Rio de Janeiro. By offering a snapshot of the application of current policies and their real impact on the health system, it aimed to identify procedural bottlenecks, differences by territory, gender, and race/color, with the expectation of expanding early reperfusion and promoting equity of outcomes.^9^

This interesting and necessary study analyzed 457 patients with acute myocardial infarction (AMI) treated in the Metropolitan Region of Rio de Janeiro, focusing on the door-to-needle time (DNT) of thrombolysis and possible regional inequalities. The analysis included sociodemographic profiles, comorbidities, reperfusion strategy, and ventricular function outcomes.

### Sociodemographic and clinical profile

The sample had a mean age of 60.4 years, with a predominance of males (72.7%). A marked racial composition was observed, with a majority of Black people, education concentrated in secondary school (38.2%), and family income between one and two minimum wages (35.8%). There was a high burden of comorbidities: hypertension (64.7%), diabetes mellitus (32%), and smoking (34.4%). This profile indicates a population with significant cardiovascular risk and potential socioeconomic vulnerability, factors that can influence both access to and quality of care. From a territorial perspective, the service areas were grouped into: North Zone/Ilha do Governador; South Zone/Grande Tijuca; West Zone; Barra da Tijuca/Jacarepaguá; Center; in addition to the macro-regions Baixada Fluminense and Metropolitan East.

### Reperfusion strategy and door-to-needle time

Regarding reperfusion, 79% (n=363) received thrombolysis; 18.5% (n=85) did not; and 2.4% (n=11) had no record. Among the 363 who received thrombolysis, 327 had a recorded apnea-thrombolysis (AT) time: the median was 77 minutes, and only 20.9% received thrombolysis within 30 minutes. This finding is clinically relevant: usual care goals recommend an DNT time of less than 30 minutes, so the median of 77 minutes suggests significant delays and opportunities for process improvement, especially in the pre-hospital stages, triage, electrocardiographic diagnosis, and medication logistics.

### Results by region

In the generalized linear model (GLM), the adjusted average DNT (min) values were: Baixada Fluminense 165.9; Barra/Jacarepaguá 124.2; Centro 67.7; Leste Metropolitano 123.5; Zona Norte 100.4; Zona Oeste 114.0; Zona Sul/Grande Tijuca 112.8. Quantile regression estimates reinforce gradients:

- Q25 (fastest service times): ~32.0 min in the South Zone/Greater Tijuca area versus 52.0 min in the Baixada Fluminense region.
- Median (Q50): 74.9 min in the Center versus 122.9 min in the Baixada Fluminense.
- Q75 (25% slower): 100.3 min in the city center versus 243.1 min in the Baixada Fluminense region.

These differences, while not always statistically significant, are clinically significant and suggest operational and access asymmetries, particularly unfavorable to the Baixada Fluminense region. The Center's performance, with systematically shorter turnaround times, may reflect greater availability of resources, more agile protocols, or better pre-hospital integration.

### Ventricular function outcome

At hospital discharge, 63.5% presented some degree of ventricular dysfunction. A trend towards a higher proportion of severe dysfunction was identified in the Barra/Jacarepaguá area, although without reaching statistical significance. Although exploratory, this finding reinforces the importance of timely care processes, given the impact of ischemia time on myocardial function.

### Comments and implications

- The low percentage of DNT ≤30 minutes (20.9%) and the median of 77 minutes point to the need for workflow re-engineering: early diagnosis (rapid ECG), screening protocols with thrombolysis triggers, prior preparation of drugs, and team training.
- The age associated with higher DNT suggests that clinical or risk assessment barriers in older patients may delay therapeutic decisions; specific interventions for this group may be beneficial.
- Regional variations, with poorer performance in the Baixada Fluminense region, indicate structural inequalities. Investments in emergency networks, regional care pathways, teleconsultation for ECGs, and standardization of protocols can reduce disparities.
- The combination of GLM and quantile regression is methodologically appropriate for asymmetric data, allowing the capture of differences in both the center and the tails of the distribution.

### Limitations (inferred)

Incomplete records (e.g., missing DNT in some thrombolyzed patients and reduced N in the GLM group) may introduce selection bias. The absence of significance in some analyses does not exclude clinical relevance, especially given the observed effect sizes and gradients.

## Conclusion

A high burden of cardiovascular risk was observed in a population with socioeconomic vulnerabilities. Despite the widespread use of thrombolysis, post-acute thrombolysis (PAT) proved to be prolonged and heterogeneous across regions, with consistently lower performance in peripheral areas. Prioritizing strategies focused on territorial equity is fundamental to improving outcomes, including left ventricular function after an acute event.
